# BuyangHuanwu Decoction attenuates cerebral vasospasm caused by subarachnoid hemorrhage in rats via PI3K/AKT/eNOS axis

**DOI:** 10.1515/biol-2022-0071

**Published:** 2022-07-13

**Authors:** Weiping Li, Ru Wang, Wei Huang, Yanfang Shen, Jumei Du, Ye Tian

**Affiliations:** The College of Life Sciences, Northwest University, Xi’an 710069, Shaanxi, China; Department of Neurology, The Second Affiliated Hospital of Shaanxi University of Chinese Medicine, Xianyang 712000, China; Department of Science and Technology Education, Dongguan Kanghua Hospital, Dongguan 523000, China; Department of Psychiatry and Psychology, No. 923 Hospital of Joint Logistic Support Force of Chinese People’s Liberation Army (PLA), Nanning, 530000, China; Department of Medical Research Center, Northwest University Affiliated Hospital/Xi’an No. 3 Hospital, Xi’an 710016, Shaanxi, China

**Keywords:** BuyangHuanwu Decoction, subarachnoid hemorrhage, cerebral vasospasm, NO, eNOS, PI3K/AKT

## Abstract

The ancient Chinese remedy BuyangHuanwu Decoction (BHD) is used to treat qi deficit and blood stasis conditions. This work investigated the effect of BHD on cerebral vasospasm (CVS) caused by subarachnoid hemorrhage (SAH). Rats were randomly assigned into four groups: control group, SAH group, SAH + BHD [13 g/(kg day)] group, and SAH + BHD [26 g/(kg day)] group. The Garcia neurological scoring scale was used to assess neurological dysfunction. Hematoxylin and eosin stains were used to determine the extent of vasospasm by measuring the diameter of the basilar artery. Western blot was used to measure the concentrations of phosphoinositide 3-kinase (PI3K), AKT, and phospho-AKT expression levels. RT-PCR was used to determine PI3K and AKT RNA expressions. Immunohistochemistry and enzyme-linked immunosorbent assay were used to measure levels of endothelial nitric oxide synthase (eNOS) and nitric oxide (NO), respectively, in cerebrospinal fluid. BHD treatment ameliorated CVS and mitigated neurological dysfunction after SAH. Furthermore, the findings suggest that NO concentration was increased through the activation of classical PI3K/AKT signaling and the eNOS pathway. Thus, BHD showed multifaceted roles in preventing damage via decreasing vasospasm and improving neurological impairments caused by CVS after SAH.

## 1 Introduction

According to existing registration data, cerebrovascular disease is the leading cause of death. In brief, blood vessels turned in narrow in the brain region, consequently leading to the arrest of blood flow to the brain tissue. Thereby, a lack of oxygen or nutrient is formed. Continuously, it leads the cell death or tissue damage in the brain called cerebral vasospasm (CVS) [1]. CVS is caused by inflammation, oxidative stress, brain damage, and other processes after subarachnoid hemorrhage (SAH) and can lead to recurrent stroke [2]. The pathogenesis of CVS after SAH includes the imbalance between vasoconstricting and vasodilating substances and nerve and mechanistic changes that regulate vascular tone, which may also be the primary predictor of mortality in patients with CVS [3]. In Chinese medicine, both ischemic and hemorrhagic strokes involve qi deficiency and blood stasis syndrome, manifesting as shortness of breath, dizziness, lack of spirit, fatigue, pale tongue, and nerve dysfunction [4,5].

BuyangHuanwu Decoction (BHD) is a traditional Chinese medicine used to strengthen and activate blood [5]. The components of BHD include the medicinal herbs Huangqi (*Radix Astragali Seu Hedysari*), Danggui (*Radix Angelica Sinensis*), Chi Shao (*Radix Paeoniae Rubra*), Chuanxiong (*Rhizoma Ligustici Chuanxiong*), Honghua (*Flos Carthami*), Taoren (*Semen Persicae*), and Dilong (*Pheretima*) [6]. BHD is widely applied in Chinese cerebrovascular diseases and acts on the entire neurovascular unit. It is a multi-target neuroprotective drug that acts on vascular endothelial cells and regulates cerebral blood flow [7]. In addition, BHD is commonly used for the treatment of ischemic stroke, and its effects include protecting the nervous system, promoting peripheral nerve regeneration, improving neurological function recovery, reducing infarct volume, and repairing injured blood vessels and diseased tissue, with good clinical efficacy and safety [8]. However, clinical evidence for BHD treatment in individuals suffering from hemorrhagic stroke remains relatively scarce. Modern Chinese medicine believes that preventing CVS may play a positive role in SAH prognosis [9,10,11]. The combined use of cerebrospinal fluid (CSF) replacement and BHD treatment, used in Western medicine and Chinese medicine, respectively, for SAH treatment could help lower the number of people who get CVS in later stages of life.

However, the mechanism of action for relieving CVS has not been explored [12]. Therefore, this study focused on the protective effect of BHD on CVS caused by SAH in model rats, as well as potential mechanisms.

## 2 Materials and methods

### 2.1 Main materials and regents

The Second Affiliated Hospital of Shaanxi University of Chinese Medicine Pharmacy created the BHD. *Radix Astragali* (Huangqi), *Radix angelicaesinensis* (Danggui), *Radix paeoniaerubra* (Chishao), *Rhizoma ligustici chuanxiong* (Chuanxiong), *Semen persicae* (Taoren), *Flos carthami* (Honghua), and *Lumbricus* (Dilong) were prepared by decoction in water and alcohol, as 5.0 g/mL extracts. Primary antibodies targeting phosphoinositide 3-kinase (PI3K) (cat. No. AF3241), p-Akt (cat. No. AF0908), and endothelial nitric oxide synthase (eNOS) (cat. No. GB11086) and secondary antibodies horseradish peroxidase (HRP)-labeled goat anti-mouse IgG (cat. No. GB23301) and HRP-labeled goat anti-rabbit IgG (cat. No. GB23303) were obtained from Wuhan Servicebio Biotechnology Co., Ltd. The hematoxylin and eosin (HE) staining kit (cat. No. G1120) was purchased from Beijing Soleibao Biotechnology Co., Ltd. The bicinchoninic acid (BCA) protein quantitative detection kit and the SDS-PAGE gel preparation kit were purchased from Google Biotechnology Co., Ltd. The nitric oxide (NO) enzyme-linked immunosorbent assay (ELISA) Test Kit (cat. No. A013-2) was purchased from Shanghai Qiaodu Biotechnology Co., Ltd.

### 2.2 Animals and experimental design

Our investigations used Sprague–Dawley rats (bought from the Laboratory Animal Center, Xi’an Jiaotong University, Medical Experimental Animal License Number: SCXK 2012-003). They were kept in a facility with a 12-h light–dark cycle and free access to food and water. During our investigation, 60 male Sprague Dawley rats weighing between 300 and 400 g were used.

Rats were divided into four groups: (1) control group, (2) SAH group, (3) SAH + BHD [13 g/(kg day)] group, and (4) the SAH + BHD [26 g/(kg day)] group. The dose of BHD administered was converted from that used in humans based on the rat body surface area; this dose (0.06 mL/kg) was administered twice per day for 7 days. A double-blind method was used to evaluate the behavior of the animals from all groups using the Garcia neurological scoring scale [13]. After Garcia neurological scores were measured, the animals were given a 10% chloral hydrate (350 mg/kg) anesthetic. Thoracotomy was performed on the animals, followed by perfusion. The right atrium was opened by blocking the descending aorta, and the ventricular chamber was mounted with an NO18 catheter. The animal was then perfused with 100 mL of 0.01 M phosphate-buffered saline (pH 7.4), which was followed by 100 mL of 2% paraformaldehyde in phosphate-buffered saline at 36°C and 80 mmHg pressure. The extracted brain was immersed in 2% paraformaldehyde overnight at 4°C. In all SAH animals, visual assessment confirmed that the clump had clogged the basilar artery. CSF was collected and retained for further analysis.


**Ethical approval:** The research related to animal use has been complied with all the relevant national regulations and institutional policies for the care and use of animals and was approved by the Ethics Committee of Shaanxi University of Chinese Medicine (license no: sucmdl20210305017).

### 2.3 SAH animal model and BHD treatment

Rats were anesthetized with 350 mg/kg of 10% chloral hydrate intraperitoneally. We used the upgraded double injection SAH model [14]. For the first induction of SAH, fresh arterial blood (0.1 mL/100 g) was collected from the caudal artery and administered into the cisterna magna. The mice were placed in a central position for 30 min after induction. Rats with respiratory distress were observed and, if necessary, ventilated. When they were completely awake, they were reintroduced to the veterinary house. For the second induction of SAH, animals were again injected with blood 48 h after the first SAH to delay vasoconstriction. After the model was established, the neurological function score of the SAH mice was lower than that of the control group, indicating that the model was successful.

On the second day after the model was established, two experimental groups were handled as follows: BHD was administered by continuous enema for 7 days, twice per day in the morning and evening. The equivalent dose was converted in accordance with the surface area of the experimental animals and humans using the conversion formula of the relative ratio of the body mass to the body surface area per kilogram.

### 2.4 The Garcia neurological score

The Garcia neurological scoring scale evaluated the extent of neurological impairment in six categories: rat autonomy, body symmetry, forelimb extension function, screen test, bilateral body touch, and bilateral beard reflex. A score of 18 points indicates normal function [13]. The most severe injury scored the lowest score of 3 points.

### 2.5 Basilar artery diameter analysis (HE staining)

The basilar artery was sliced into five cross-sections, and paraffin-embedded brain tissue was sliced into 25-µm-thick sections. HE staining was performed, and the tissue was observed with a video-assisted microscope. The diameter of the basilar artery was measured and analyzed with ImageJ 1.50i [15].

### 2.6 Immunohistochemical analysis

Endothelial cells were identified using video-assisted microscopy (400×). Separated rat basilar arteries were perfused and preserved with 4% paraformaldehyde. Paraffin-embedded slices of the basilar artery were treated with polyclonal anti-rabbit eNOS antibody (1:200; Servicebio, Wuhan, China) at 25°C for 40 min. Five consecutive sections of each specimen were collected, and the morphology of the lumen cross-section was analyzed.

### 2.7 ELISA analysis

An ELISA kit was used to measure the NO content of the CSF. The testing was carried out as directed by the manufacturer. As indicated in the supplier’s instructions, the upper limit of NO detected was 40 µmol/L.

### 2.8 RNA extraction and quantitative real-time PCR

A TRIzol reagent (Invitrogen, Carlsbad, CA, USA) was used to extract total RNA from brain tissue, which was then measured using a UV spectrophotometer. A SuperScript reverse transcriptase kit was used for reverse transcription (Invitrogen, Carlsbad, CA, USA). Gene expression was measured using a quantitative real-time PCR with SYBR^®^ dye (LightCycler^®^ 96 Real-Time PCR System) after the cDNA was generated. The transcription level was calculated relative to the GAPDH signal and normalized to the mean of the control group data.

The primers for PI3K, AKT and GAPDH were as follows. PI3K: forward, 5′-GGTGAGGAACGAAGAATGGC-3′ and reverse, 5′-TCCGAGGCAAGACAGGGATA-3′; AKT: forward, 5′-TGAGACCGACACCAGGTATTTTG-3′ and reverse, 5′-GCTGAGTAGGAGAACTG GGGAAA-3′; GAPDH: forward, 5′-CTGGAGAAACCTGCCAAGTATG-3′ and reverse, 5′ GGTG GAAGAATGGGAGTTGCT-3′.

### 2.9 Western blot analysis

Samples from the basilar artery (6 animals per group) were homogenized with a protease inhibitor in RIPA lysate (Servicebio) and centrifuged for 12 min at 12,000 rpm. Protein was then quantified by BCA protein quantitation (Google Biotechnology Co., Ltd.), and then, sample buffer (4×; Servicebio) was added. The samples were loaded and run on 8% SDS-PAGE gels and transferred to polyvinylidene difluoride membranes. At room temperature, membranes were blocked in 5% skim milk powder in Tris-saline with 0.2% Tween 20 (TBST). Rabbit polyclonal anti-rat PI3K (1:1000; Wuhan Servicebio Biotechnology Co., Ltd.), Akt, and p-AKT antibodies were used and anti-actin (monoclonal antibody, 1:1,000; Servicebio) served as a control. HRP-conjugated secondary antibody in TBST was added for 1 h at room temperature. SuperSignal™ West Femto Maximum Sensitivity Substrate (Thermo Fisher Scientific, Inc.) was used as a visualization substrate. Optical density was measured using a Pierce chemiluminescence image analyzer.

### 2.10 Statistical analysis

GraphPad Prism software and SPSS 25.0 (IBM Corp.) were used to analyze all of the data (version 8.2). The results were provided as mean with standard deviation. To reduce error, the trials were performed three times. To examine the differences between the groups, one-way analysis of variance was performed, followed by Tukey’s *post hoc* analysis. *P* < 0.05 was considered statistically significant.

## 3 Results

### 3.1 Establishment of the SAH animal model

**Figure 1:**
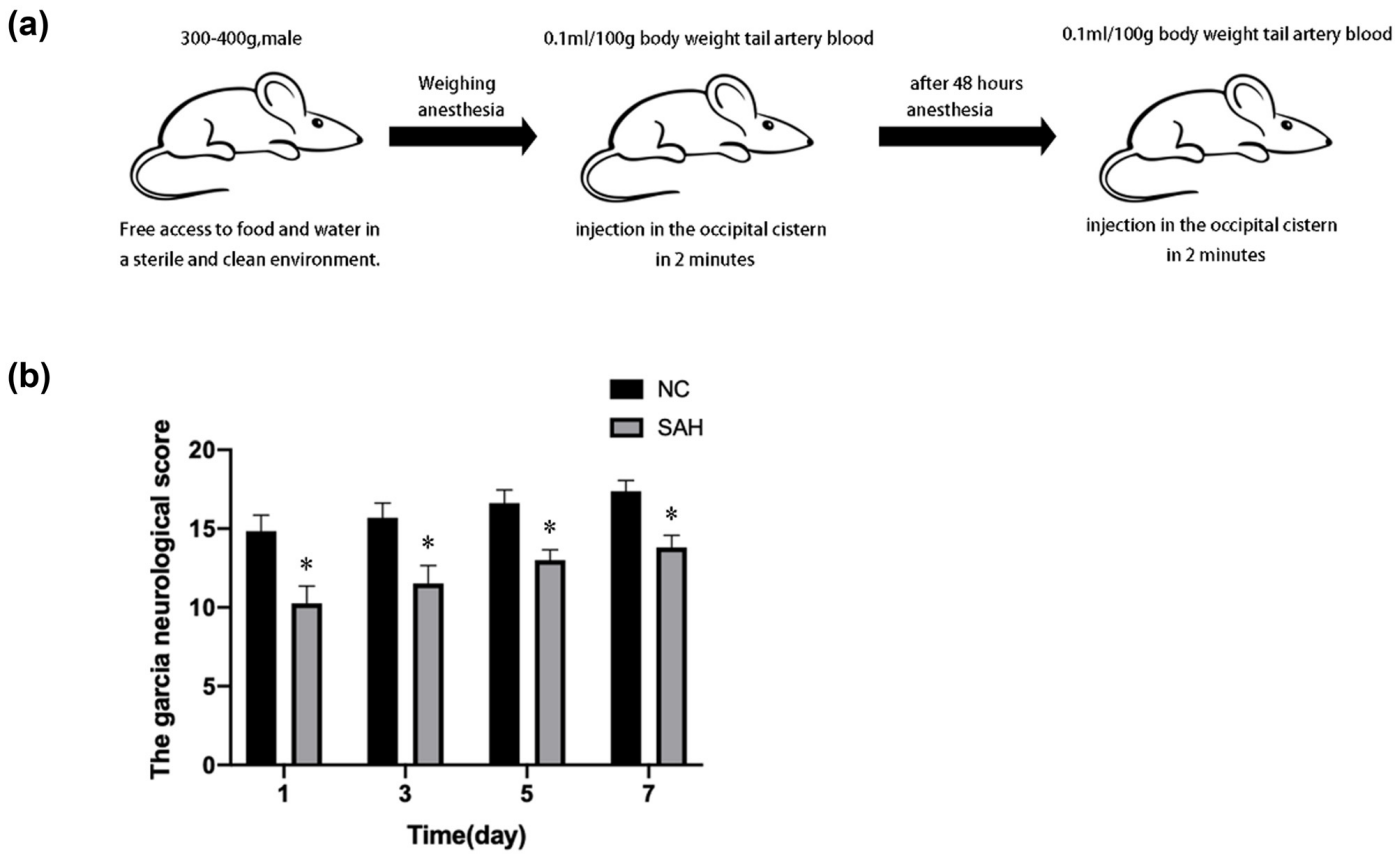
Establishment of the SAH animal model. (a) The procedure for a catheter-based double blood injection into the cisterna magna via a parieto-occipital burr hole in an experimental animal. (b) The neurological function score of the SAH group was lower than that of the control group. There was a statistically significant difference between the two groups. The data are shown as mean ± standard deviation (*n* = 5); ^*^ *P* < 0.05 is used to indicate significance.

To investigate the protective effect of BHD on CVS caused by SAH in rats, we established an SAH animal model using the improved double injection SAH model. Garcia neurological scores were measured after the model was completed. The neurological function (Figure 1a) score of SAH mice was lower than that (Figure 1b) of control group mice; when the two groups were compared, there was a significant difference (*P* < 0.01). This demonstrates that the model was successfully established.

### 3.2 BHD improved CVS-induced neurological deficits after SAH (the Garcia neurological score and the diameter of basilar artery)

**Figure 2:**
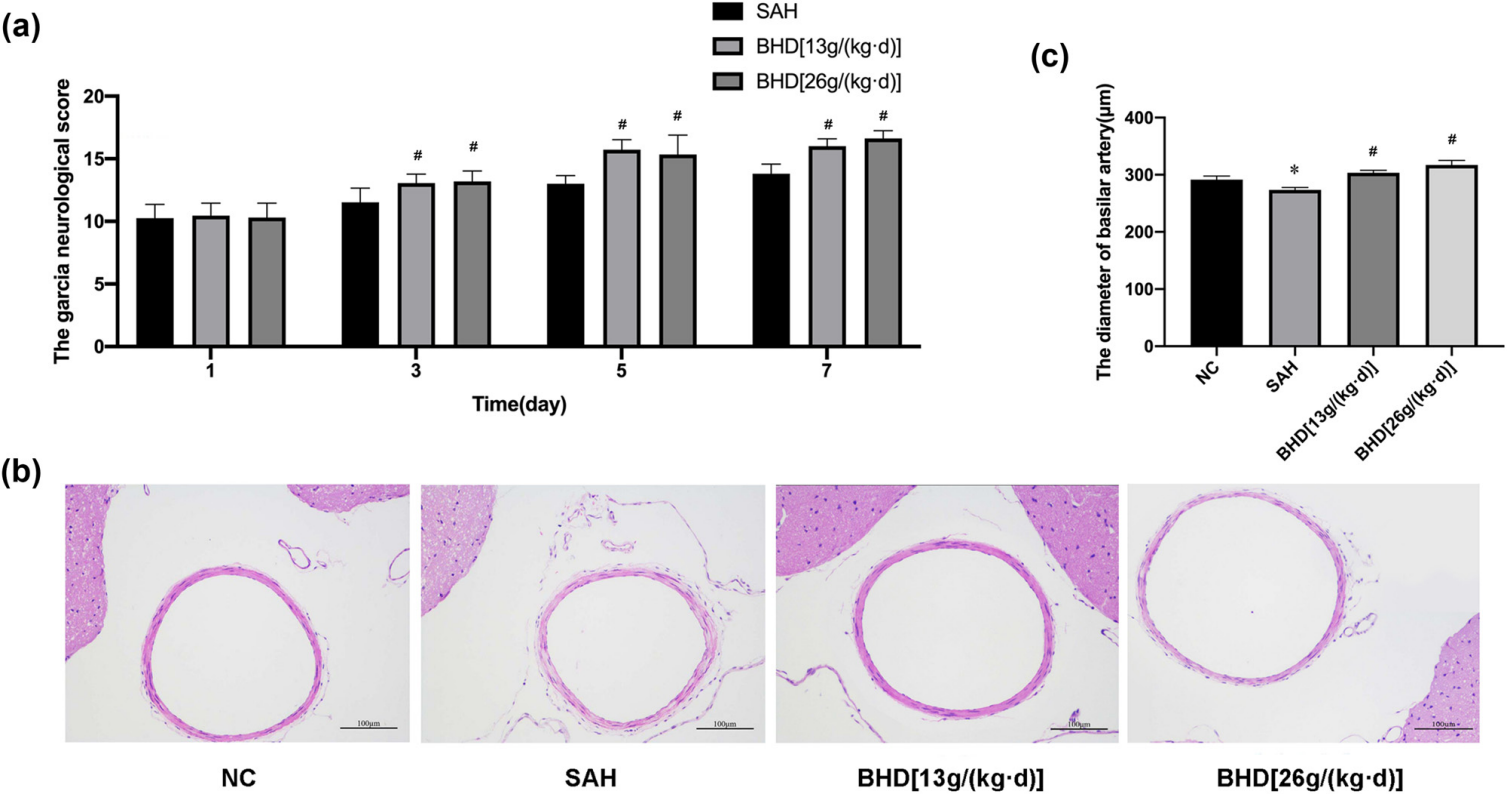
(a) BHD significantly improved the neurological score after SAH. (b) Cross-sections of the basilar artery in the different groups. (c) The diameter of the basilar artery in the SAH group was significantly reduced compared with that in the control group (^*^ *P* < 0.05 was used to indicate significance, compared with the control group). The basilar artery diameter in the SAH + BHD group was substantially larger than that in the SAH group (^#^ *P* < 0.05 was used to indicate significance, compared with the SAH group). The data were shown as mean ± standard deviation (*n* = 3).

Rats in the experimental group were treated with BHD for 1, 3, and 7 days, and neurological function scores were recorded. As demonstrated in Figure 2a, BHD considerably improved the neurological score after SAH; after 3 days of BHD treatment after SAH, the neurological function score was 14.3% higher in the BHD group than in the SAH group. After 7 days of BHD treatment, the neurological function score was 18.8% (17.0 ± 0.71 points) higher in the BHD group than in the SAH group. These results indicate that BHD improved CVS-induced neurological deficits after SAH.

Basilar artery cross-sections were stained with HE (Figure 2b). The cross-sectional area of the basilar artery (which is regarded as a useful marker for CVS following SAH) in the SAH group was reduced compared with that in the control group. The cross-sectional area of the basilar artery in the SAH + BHD group was increased after BHD treatment; the basilar artery diameter in the SAH group was 273.548 ± 4.333 µm, whereas in the control group, the SAH + BHD [13 g/(kg day)] group, and the SAH + BHD [26 g/(kg day)] group, the diameter was 291.394 ± 6.367, 303.336 ± 4.376, and 317.306 ± 7.725 µm (mean ± SD), respectively. The basilar artery diameter in the SAH + BHD group was substantially larger than that in the control group and in the SAH group. These results suggest that BHD plays a protective role in regulating vasomotor tone in CVS after SAH (Figure 2c).

### 3.3 eNOS and NO play an important role in regulating vasomotor tone in CVS after SAH

**Figure 3:**
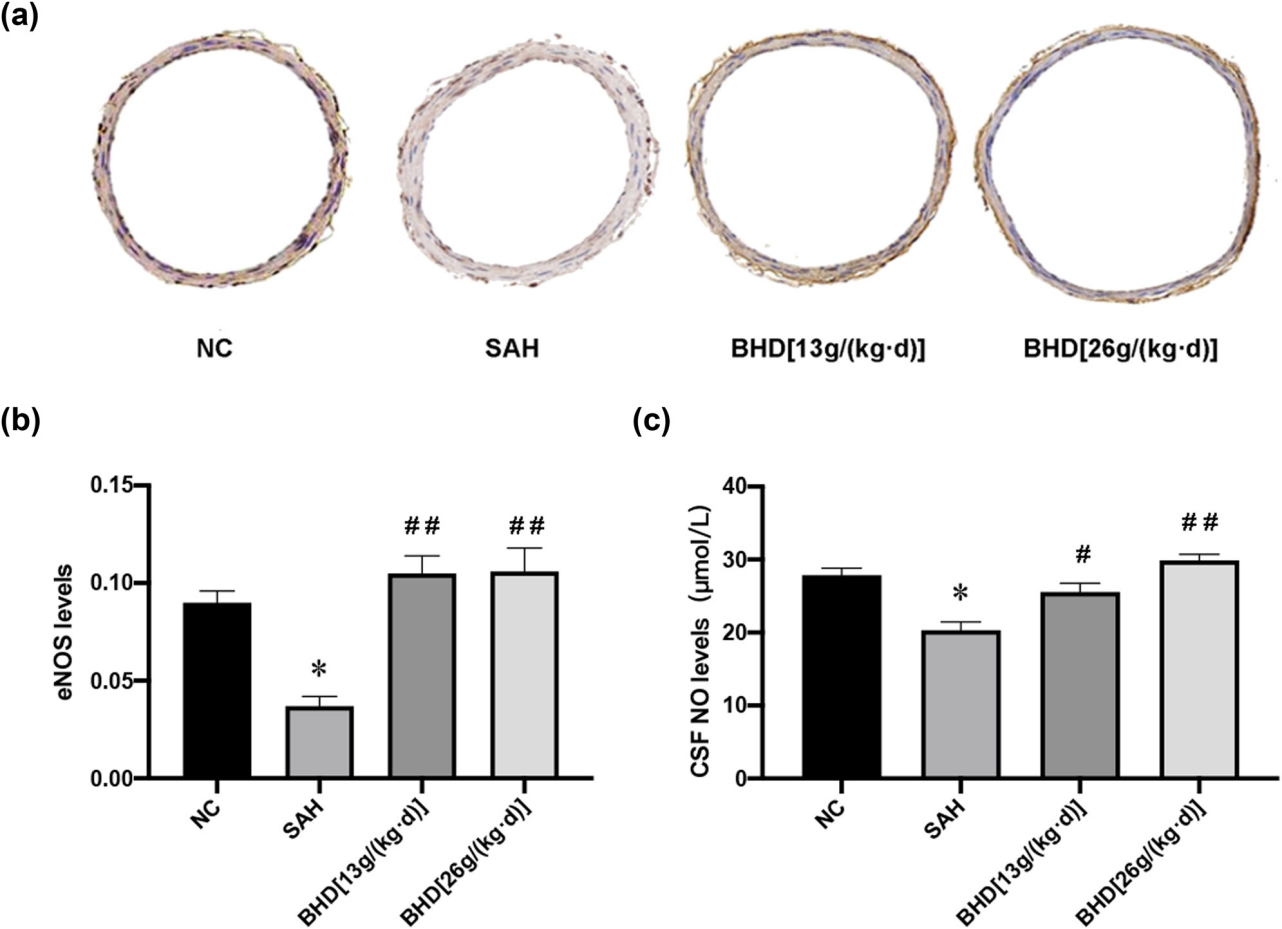
Expression of eNOS using immunohistochemistry and of NO using ELISA. (a) Immunostaining for eNOS in basilar artery cross-sections from different groups. (b) The eNOS level in the SAH group was significantly lower than that in the control group (^*^ *P* < 0.05 was used to indicate significance, compared with the control group). BHD significantly increased the expression of eNOSafter SAH (^##^ *P* < 0.01 was used to indicate significance, compared with the SAH group). The data were shown as mean ± standard deviation (*n* = 3). (c) The concentration of NO in the CSF in the SAH group was considerably lower than that in the control group (^*^ *P* < 0.05 was used to indicate significance, compared with the control group). BHD significantly increased the expression of NO after SAH (^#^ *P* < 0.05 and ^##^ *P* < 0.01 were used to indicate significance, compared with the SAH group). The data were shown as mean ± standard deviation (*n* = 3).

Immunohistochemistry analysis showed higher protein levels of eNOS in the control group (Figure 3a). Compared with the SAH group, the SAH + BHD group had increased expression of eNOS proteins (Figure 3b). NO levels in the CSF were measured using an ELISA kit that was purchased commercially, and it was found that the concentration of NO in the CSF of the SAH group was considerably lower than that in the control group (Figure 3c). In the SAH rats, BHD raised NO levels in the CSF. In the SAH group, the baseline NO level was 20.32 ± 1.13 mol/L. After 7 days of treatment with BHD, the level of NO was significantly increased compared with the SAH group. These results suggest that BHD reverses the NO decrease in CSF.

### 3.4 BHD significantly upregulated the PI3K/AKT signaling pathway

**Figure 4:**
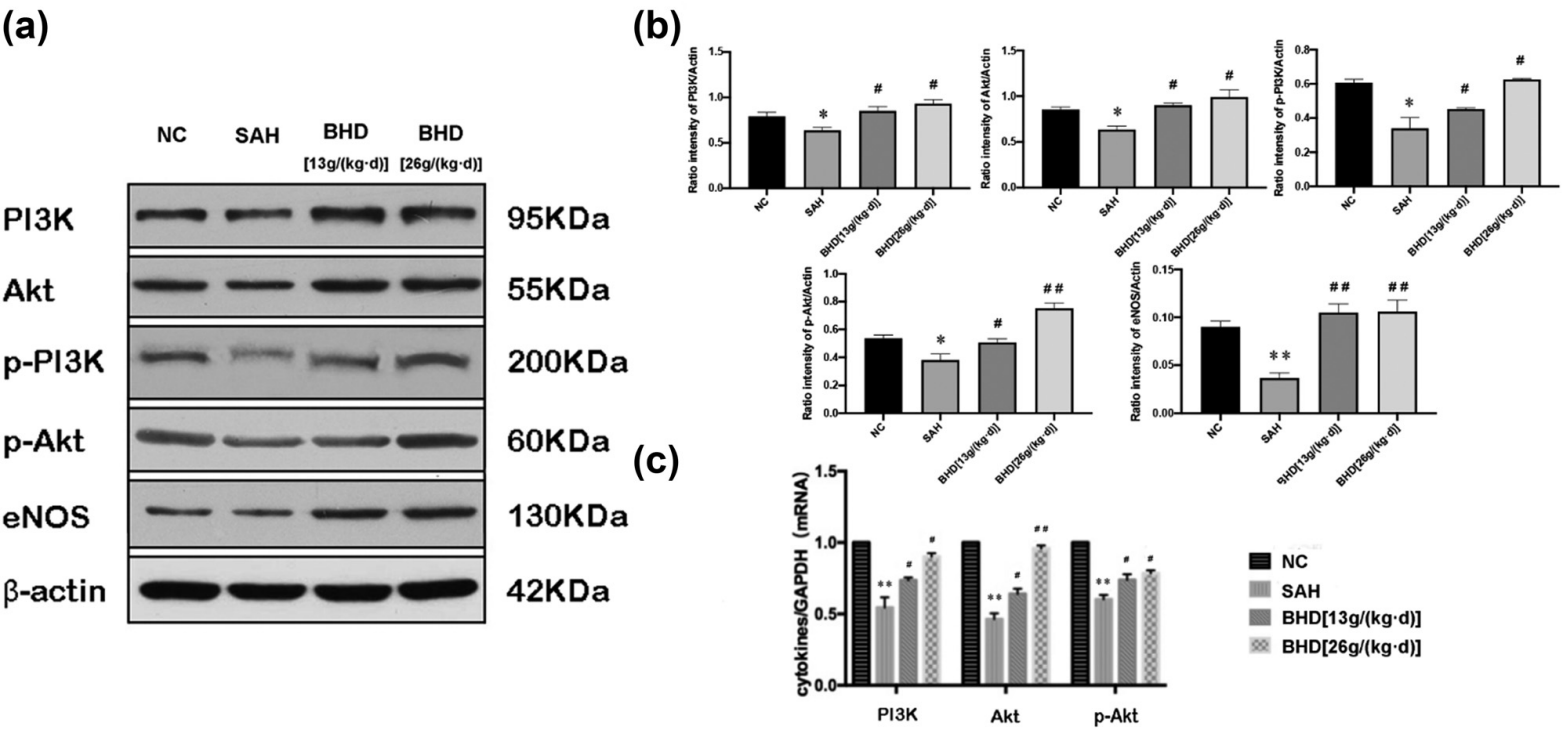
Analysis of PI3K, AKT, and p-AKT expression levels by western blot and quantitative real-time PCR. (a) The blots showed that the SAH group had lower levels of PI3K, AKT, and p-AKT than the control group, and the graphs (b) show the ratio of expression compared with actin. BHD increased the expression of PI3K, AKT, and p-AKT as compared with the SAH group. (c) Quantitative real-time PCR analysis of PI3K, AKT, and p-AKT. Quantitative real-time PCR analysis of data was adjusted for GAPDH mRNA content (*n* = 3) and examined in three separate quadruplicate experiments; data were shown as mean ± standard deviation (*n* = 3); ^*^ *P* < 0.05, compared with the control group and ^#^ *P* < 0.05, compared with the SAH group, were used to indicate significance.

We found a reduction in PI3K, AKT, and p-AKT in the SAH group compared with the control group at both the protein level (Figure 4a) and transcription level (Figure 4c). The levels of PI3K, AKT, and p-AKT in the SAH + BHD group were substantially higher than those in the SAH group (*P* < 0.01) (Figure 4). These findings indicate that BHD induced the expression of PI3K, AKT, and p-AKT and suggest that BHD alleviated CVS and reduced the recurrence of CVS after SAH by significantly upregulating the PI3K/AKT signaling pathway.

## 4 Discussion

We found that BHD had anti-vasospasm effect in SAH animals via stimulating the PI3K/AKT/eNOS signaling pathway and reducing vasoconstriction. The anti-vasospasm effect of BHD appeared to be accompanied by regulation of eNOS and NO production.

NO is a gaseous free radical that relaxes blood vessels through the regulation of cyclic guanosine monophosphate-dependent or -independent pathological mechanisms that regulate the release of other vasoactive substances (such as endothelin-1 and prostacyclin), as well as through direct activation of potassium channels [16,17]. Smooth muscle cellular membrane hyperpolarization and calcium channel activation in the sarcoplasmic reticulum lower intracellular Ca^2+^ concentrations, resulting in the relaxation of smooth muscles [18,19,20]. After SAH, blood clots in the subarachnoid space break apart and release products that induce inflammatory and stress responses, reducing NO synthesis in the vascular wall and thus reducing NO-dependent vasodilation. Decreased regulation leads to a pathological contraction of the vessel wall, and CVS occurs. The nitrogen atoms and oxygen molecules at the terminus of the l-arginine oxime catalyzed by eNOS and neuronal nitric oxide synthase (nNOS) are the chief sources of NO engaged in cerebral blood supply control. The disappearance of nNOS activity in the outer membrane makes eNOS a key factor affecting NO synthesis. As a result, boosting eNOS catalytic activity and gene expression is a popular strategy for preventing and controlling CVS [21].

PI3K/AKT is a crucial upstream molecule that controls eNOS activity and expression. PI3K translocases inactive cytoplasmic AKT to the cell membrane, where it is activated to dephosphorylate the eNOS Thr495 residue linked to calmodulin on the cell membrane, resulting in eNOSactivation [22]. BHD has been previously shown to significantly improve the neurological scores of rats with intracerebral hemorrhage, reduce blood–brain barrier permeability and cerebral edema, activate the PI3K/AKT signaling pathway, and significantly upregulate p-AKT expression, and thus reduce brain damage caused by cerebral hemorrhage [23]. Other research showed that BHD activated thrombopoietin receptor-2 by activating PI3K/AKT signaling and thereby promoted the formation of intracranial neovascularization in an intracerebral hemorrhage rat model [24]. BHD treatment can also upregulate the activity related to the vascular endothelial growth factor receptors Flk-1 and Flt-1 and can improve hemodynamic function in rats with cerebral hemorrhage, which might be related to its regulation of blood vessel regeneration and its promotion of loss of regional microvascular remodeling [25]. BHD increased the transcription of Ang-1 and Tie-2 mRNA in the brain of rats with intracerebral hemorrhage, enhanced microvessel rebuilding in the affected site, and aided tissue repair [26]. In the current investigation, BHD increased the expression of PI3K and p-AKT in SAH mice, possibly enhancing its vasodilatory action and activating NO generation by eNOS. Currently, treatment of SAH induced by cerebral aneurysm and concomitant vasospasm is disappointing [27,28]. Patients with vasospasm have higher hospitalization costs and longer hospital stays and lack effective medical care [29]. Clinically, oral administration of nimodipine may improve functional outcomes in patients with SAH, but the efficacy for improving vasospasm is inaccurate [30,31]. Here, we have shown that BHD effectively activated the PI3K/AKT pathway and increased eNOS expression.

## 5 Conclusion

Our findings showed that BHD administration was a harmless and viable strategy to avert CVS in this experimental setting. Its anti-vasospasm impact may be aided by eNOS, PI3K, and AKT, in addition to increased NO levels. The findings indicate potential treatment effects of this Chinese herbal medicine against arterial spasms after SAH.
